# EUBUCCO v0.1: European building stock characteristics in a common and open database for 200+ million individual buildings

**DOI:** 10.1038/s41597-023-02040-2

**Published:** 2023-03-20

**Authors:** Nikola Milojevic-Dupont, Felix Wagner, Florian Nachtigall, Jiawei Hu, Geza Boi Brüser, Marius Zumwald, Filip Biljecki, Niko Heeren, Lynn H. Kaack, Peter-Paul Pichler, Felix Creutzig

**Affiliations:** 1 Mercator Research Institute of Global Commons and Climate Change, Berlin, 10829 Germany; 2 Technical University Berlin, Berlin, 10623 Germany; 3 Independent researcher, Berlin, 12053 Germany; 4 ETH Zürich, Institute for Environmental Decisions, Zürich, 8092 Switzerland; 5 National University of Singapore, Singapore, 119077 Singapore; 6 Norwegian University of Science and Technology (NTNU), Trondheim, 7491 Norway; 7 Hertie School, Data Science Lab, Berlin, 10117 Germany; 8 Potsdam Institute for Climate Impact Research (PIK), Potsdam, 14473 Germany

**Keywords:** Sustainability, Geography, Climate-change mitigation, Climate-change policy

## Abstract

Building stock management is becoming a global societal and political issue, inter alia because of growing sustainability concerns. Comprehensive and openly accessible building stock data can enable impactful research exploring the most effective policy options. In Europe, efforts from citizen and governments generated numerous relevant datasets but these are fragmented and heterogeneous, thus hindering their usability. Here, we present eubucco v0.1, a database of individual building footprints for ~202 million buildings across the 27 European Union countries and Switzerland. Three main *attributes* – building height, construction year and type – are included for respectively 73%, 24% and 46% of the buildings. We identify, collect and harmonize 50 open government datasets and OpenStreetMap, and perform extensive validation analyses to assess the quality, consistency and completeness of the data in every country. eubucco v0.1 provides the basis for high-resolution urban sustainability studies across scales – continental, comparative or local studies – using a centralized source and is relevant for a variety of use cases, e.g., for energy system analysis or natural hazard risk assessments.

## Background & Summary

Built infrastructure fulfills the basic need for shelter and mediates access to fundamental infrastructural services for the population^1^. The economic value of global real estate in 2020 was estimated to $327 trillion, nearly four times the global gross domestic product^2^. Built infrastructure accounts for the majority of societies’ physical material stock: in particular, building construction and maintenance is responsible for half of global resource consumption^3^. Buildings also account for a substantial share of the global final energy consumption and greenhouse gas emissions, respectively 31% and 21% in 2019^4^. The way we build significantly affects material and energy consumption and associated greenhouse gas emissions and other impacts^4^. High-resolution building stock data can support economic and social policy at the regional level, especially to achieve the Sustainable Development Goals^5,6^.

The highest resolution building stock data that is typically used is geospatial vector data. At a minimum, they contain georeferenced two-dimensional (2D) *footprints* of individual buildings and can reach a realistic 3D representation of walls, roof and further details. Geospatial vector building *geometries* are enriched with numerical and categorical *attributes* that include the building *height* (also known as 2.5D representation), *construction year*, and usage *type*. Other potential *attributes* can be information about retrofitting, roof type, energy standards, building materials, etc. In contrast to aggregate data, such high-resolution building stock data allow to consider buildings individually and collectively for planning policy interventions. By correlating building *attributes* with one another and accounting for spatial context, these data allow for more targeted analyses and maps that relate building *attributes* to demographic information, help design targeted policy interventions, and model their impacts down to the building level.

In regional planning, high-resolution building stock data enables to investigate different important questions. They are necessary to assess future demand for new construction, as well as possible needs for deconstruction in areas with shrinking populations^7–9^. Building stock composition and dynamics can also serve as a basis for predicting material outflow either as waste or as raw material for new building construction^10–13^. In turn, in energy and climate policy, spatially resolved data on the extent and condition of the building stock is essential for modeling energy demand scenarios and climate change policies aimed at reducing energy-related greenhouse gas emissions^14–16^. Finally, this information can be used in risk models for natural hazards or economic damage functions related to climate change, where it enables an explicit representation of the exposure of a building stock.

Europe offers unique conditions to prototype a database of building stock characteristics at the continental level, as there are several country-wide datasets (e.g., Spain, France, Netherlands) with a joint availability of *footprints* and *attributes*. However, there is currently no single database combining all buildings and relevant attributes digitally available in Europe. Tools developed by the European Union (EU) like CORDA^17^ or EU Building Stock Observatory^18^ represent first attempts at creating such database, but the first focuses on the seamless integration of only few datasets with highest quality standards, while the second only provides country-level aggregated statistics. There has been a trend towards more open data releases from governments in recent years, partly orchestrated by the European Union project INSPIRE^19,20^. Unfortunately, these numerous datasets are fragmented and heterogeneous, which hinders their usability. In Europe, OpenStreetMap (OSM) or Microsoft^21^ are the only sources for harmonized building data for all EU countries. The former is based on the contributions of millions of mappers^22–24^, yet has quality issues such as varying coverage, inconsistent description, and lack of *attributes*^24^. The latter is derived from remote sensing data and, hence, lacks spatial accuracy and climate relevant building attributes. Therefore, there is a need to identify available data, assess their quality, and aggregate the best existing datasets to create a complete building database for Europe. Such a centralized database can amplify the value of individual datasets by unlocking novel research opportunities across scales^16,25–27^, including comparative studies such as city typologies^27^ and unprecedented continental-level studies.

**Fig. 1:**
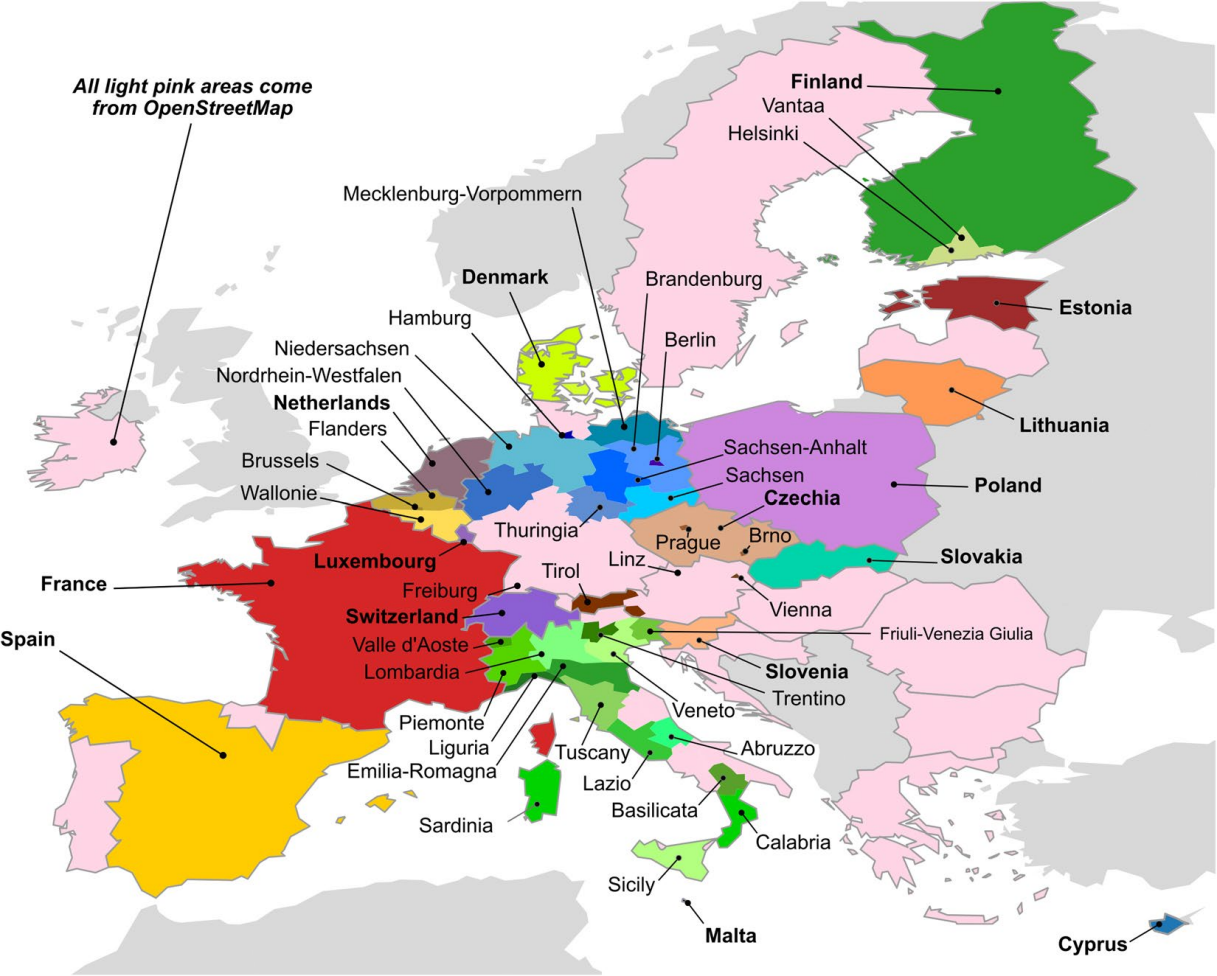
The 50 input datasets parsed to generate eubucco v0.1. Bold font indicates country-level datasets, while normal font indicates region- or city-level datasets. Datasets for a same country are designated with different tones of the same color. All areas where OpenStreetMap was used as basis for the building footprints are colored in light pink.

Here, we present eubucco v0.1^28^, a database of individual buildings covering all 27 EU countries and Switzerland, which represent 378 regions and 40,829 cities. eubucco v0.1 contains building *footprints* for ~202 million buildings and three main building *attributes* – *height*, *construction year* and *type* – for respectively 73%, 24% and 46% of the buildings, see Table 1 for country-level values. Our input datasets include 50 heterogeneous open government datasets and OSM, see Fig. 1. Our workflow involves three steps. First, we identified candidate datasets. Second, we retrieved data involving negotiation, web scraping, and various application programming interfaces (API). Third, we harmonized the data to make *geometries* and *attributes* comparable. The last step also involved introducing a consistent administrative sub-division scheme that underpins the database structure. Finally, we performed extensive validation to monitor data coverage and quality throughout our workflow.

**Table 1:** Country-level content statistics of eubucco v0.1.

Country	Buildings [*n*]	Footprint area [m^2^]	Heights [%]	Ages [%]	Types [%]
Germany	43,644,887	6,108,343,562	66	0	66
France	47,847,462	5,851,128,661	98	45	54
Italy	20,674,153	3,668,104,389	69	7	50
Spain	16,340,067	2,979,509,619	95	99	100
Poland	14,404,767	2,099,046,447	100	0	0
Netherlands	9,692,657	1,202,665,089	100	100	0
Belgium	8,634,500	1,096,372,592	100	0	0
Austria	4,135,733	867,271,697	7	0	16
Denmark	5,691,756	738,533,831	0	0	0
Finland	5,370,223	691,145,893	2	1	1
Czechia	4,044,659	673,376,186	8	0	92
Sweden	2,532,313	568,473,803	3	0	27
Switzerland	2,641,571	506,395,304	100	0	0
Slovakia	3,488,125	428,026,555	95	0	87
Hungary	1,546,359	337,332,876	3	0	33
Portugal	1,215,018	325,743,275	4	0	32
Romania	1,332,570	323,929,666	7	0	21
Lithuania	1,924,431	290,400,677	0	0	0
Ireland	1,610,614	243,282,004	13	0	67
Greece	864,237	187,420,816	5	0	13
Slovenia	1,162,832	182,617,285	94	0	19
Croatia	873,080	147,590,430	1	0	19
Bulgaria	448,470	145,130,155	15	0	35
Estonia	803,218	132,899,346	100	0	0
Latvia	513,316	112,759,248	6	0	17
Cyprus	467,594	74,417,048	100	0	0
Luxembourg	143,923	43,143,729	100	0	0
Malta	142,616	32,599,347	100	0	0
**Total**	**202,191,151**	**30,057,659,533**	**73%**	**24%**	**46%**

Values correspond to the final counts from the final provided as the database at the end of pipeline, and do not account for the buildings that were dropped throughout the workflow. The values for *height*, *construction years* and *types* correspond to the percentage of buildings for which the attribute is available in eubucco v0.1. Countries are ordered by descending total building footprint area.

eubucco v0.1 gathers timely information for high-resolution analysis of the EU building stock, which is highly relevant for policy making on the EU, national and city level, urban planning as well as academic research. By identifying and assessing the relevance of various datasets, we enable users to easily find and access data across the EU. By collecting, harmonizing, cleaning and redistributing all the data through a simple download approach, we ensure a high usability. The data is available on the dedicated website eubucco.com and referenced on Zenodo^28^ (concept DOI: 10.5281/zenodo.6524780, v0.1-specific DOI 10.5281/zenodo.7225259). The code used to generate the database is provided as a Github repository^29^, with tags that correspond to a specific version, e.g., v0.1 together with the documentation of all input data to enable transparent re-use, verification, update and modification. This database is therefore reproducible, i.e., the code allows to recreate the repository with little manual intervention.

## Methods

**Fig. 2:**
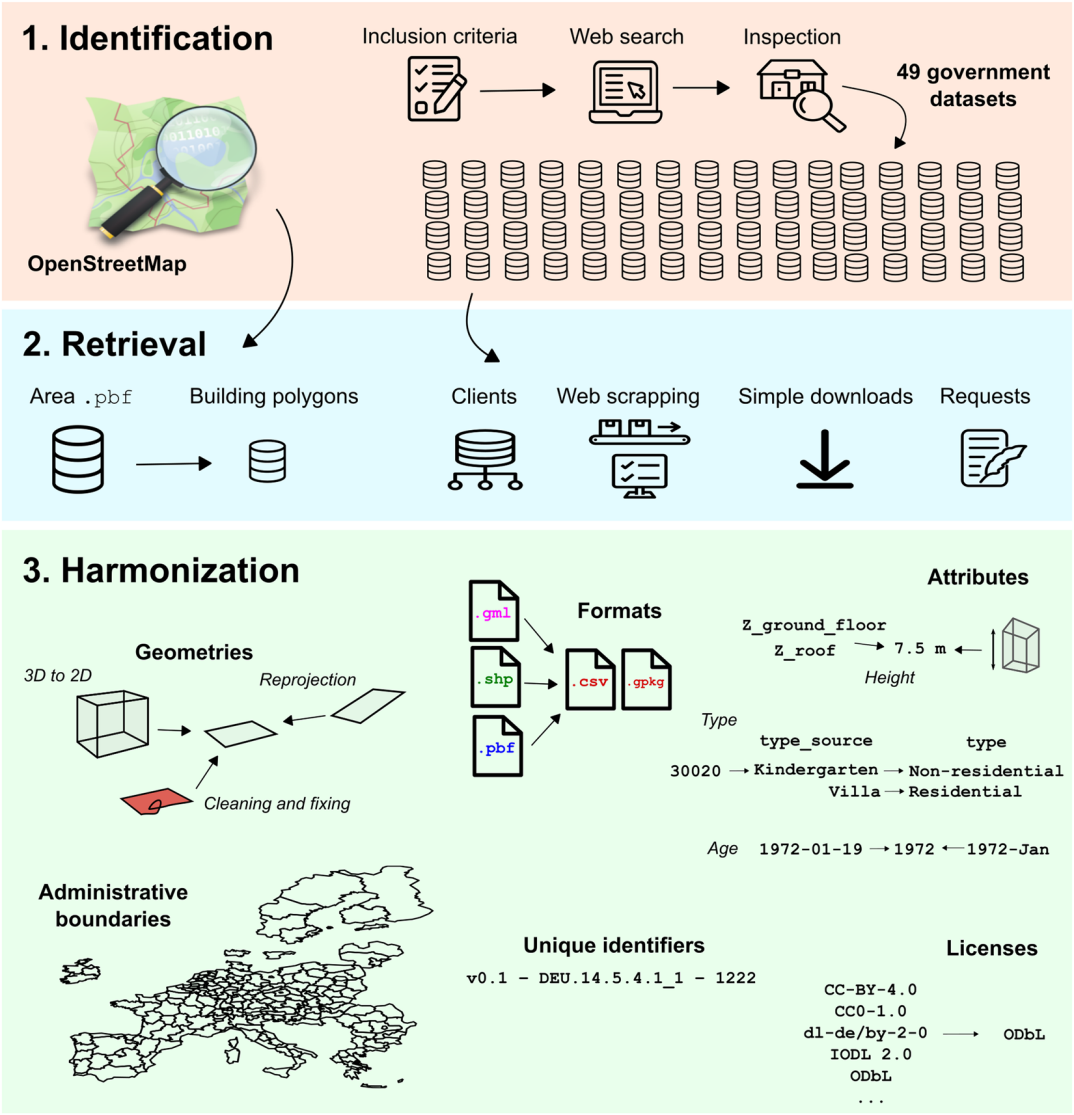
Overview of the processing workflow of eubucco v0.1.

Creating eubucco v0.1 involved three main steps: 1) identifying relevant data; 2) retrieving it from individual websites; and 3) harmonizing the various input datasets into one common format with consistent building *footprint geometries*, *attributes* (*height*, *type* and *construction year*) and administrative boundaries (country, region and city) to create a database structure. We performed extensive data validation procedures throughout the workflow to guarantee completeness, minimal errors and no duplicates (see Technical Validation). The different steps of the workflow are summarized on Fig. 2.

Our data processing workflow^29^ is almost entirely written in Python in order to maximise automation and reproducibility compared to desktop geographic information system (GIS) software. We created a Python module eubucco with core functions for each of the processing steps. In order to facilitate updates once new data become available, whenever possible we wrote generic functions that for most steps can be run in parallel for each datasets via an argument parser, e.g., as a Slurm job array. We also used PostGIS and QGIS for a small number of tasks, documented on the repository, e.g., via .txt files.

### Data identification

eubucco v0.1 contains 50 individual datasets, which we first had to identify and screen for inclusion. See a detailed summary of all the input datasets used in input−dataset−metatable−v0.1.xlsx in Data Records. Refer to the upper panel of Fig. 2 for a visualization of the data identification steps within the workflow.

#### Inclusion criteria

The buildings in eubucco v0.1 are defined liberally as a*ny permanent structure with a roof and walls*. As criterion for inclusion, only datasets containing geospatial vector data of the *footprint* – or in other words, the ground surface – were considered. Input datasets can either be 2D (with only the *footprint* as *geometry*), 2.5D (with *footprint* and *height* as one or several *attributes*, e.g., max height, height of the eaves, etc.) or 3D (with walls and roof *geometries*). One-point coordinate data, for example in an address dataset, is considered as not sufficient for inclusion and a polygon representation is required. Input datasets ideally contain *attributes* of interest (*height*, *construction year* or *type*), but this was not a requirement. The dataset coverage can be at country, region or city level. We did not set inclusion criteria related to the publication date of the dataset. In cases when several versions of the dataset existed, we used the latest. We acknowledge that our dataset does not represent a snapshot of the EU building stock at a given moment, but rather contains the newest data available for each area to the best knowledge of the authors. Finally, the license under which the dataset was originally released at least has to allow free use for scientific research, see detailed license information in input−dataset−metatable−v0.1.xlsx. We included datasets that did not allow for redistribution or commercial use but such datasets were treated separately, see the Outbound licensing section for details.

#### Search approach

For OSM, no search was needed given that OSM is a single dataset. For government sources, we first searched for country-level datasets. When none were found, we searched for region- and city-level datasets. We used the geoportal INSPIRE^19^, and screened entries in the spatial data theme ‘Buildings’. We queried a standard search engine, and country-specific open data portals for ‘building dataset’ in national languages. We also used technical keywords specific to this kind of datasets, e.g., ‘LoD1’ – the *level of detail* of a 3D building dataset^30^. Finally, we found additional datasets by crowdsourcing on social media.

Through this approach we could identify 49 relevant datasets, but also countries and regions where government datasets exist but are not open or only available for a fee, often prohibitively high. In such cases, we contacted the relevant data owner to ask whether the data could be used for academic purposes. See excluded−datasets.xlsx for the list of relevant datasets identified that were not included with the reasons for excluding them. The table includes the contact date, as the license of a given dataset may change in the future and become open.

#### Selecting between relevant datasets

Whenever possible, we favored government data over OSM as basis for the *footprints*. The rational for this choice is that, when available, government data tends to have a better coverage both in terms of *footprints* and *attributes* than OSM, even if the opposite can happen^31,32^. A future version of this database should include a detailed comparison of OSM and government data. When no data for a country or regions of a country was available, OSM was the fallback for building *footprints* in eubucco v0.1. In some cases, several candidate datasets representing the same area were found on a regional open data portal with a description and metadata that was not conclusive; then, we analyzed data samples of each candidate to determine which one to include. If an area is available in an individual dataset but also part of a larger dataset, different inclusion decisions were made. If the smaller dataset did not contain additional *attributes* of interest (*height*, *construction year* or *type*) compared to the larger one, then the larger dataset is being used. If the smaller dataset contains any *attributes* but the larger area does not, then the smaller dataset is used for this area. For Prague and Brno in Czechia, we had to make an arbitrary decision between the country-level data that contained *type* information and the city-level datasets that contained *heights* – here we opted for *height*.

### Data retrieval

Once identified, retrieving the data involved the download of the relevant files via various interfaces on government portals, as well as downloading OSM data from the Geofabrik server. We retrieved in total 190,387 individual files for the 50 datasets. Refer to the middle panel of Fig. 2 for a visualization of the data retrieval steps within the workflow.

#### Government data

A large heterogeneity exists in term of download services for government datasets: selection tools on interactive maps, few to many links on simpler or more complex web pages, and APIs. This required domain knowledge of each specific approach and sometimes required to build dataset-specific web scraping routines. The download approach for each dataset is documented in input−dataset−metatable−v0.1.xlsx.

Datasets are provided either as one file, multiple files corresponding to smaller administrative areas or tiles, and sometimes several levels of aggregation are available. If the data could be downloaded from a government portal via few single links or queried generating download links via email, the download was conducted manually.

In cases where a high number of links were present or a complex and time consuming download procedure was required, we used Python web scraping tools to automatically download the data, see database/preprocessing/0−downloading^29^. We developed specific download workflows for 10 different websites, building up on the web scraping packages Selenium^33^.

In a few cases, we downloaded the data via APIs or transfer protocols, including WFS, OGC API, and FTP. When datasets where available as ATOM feeds, we used web scrappers instead of browser-based clients. In three cases, the datasets were only available via a selection tool on an interactive map with low limits per query, making the manual download of the whole area virtually impossible. In such cases, we contacted the data owner to ask for a data dump, which they did provide in the cases of Emilia-Romagna and Piemonte, while Niedersachsen provided a URL to a list of download links.

#### OpenStreetMap

OSM data was downloaded as .pbf files from the Geofabrik download server^34^ using the Python library Pyrosm^35^, either at the country level or region level for large countries such as Germany and Italy where regional downloads are possible. The retrieval of buildings from OSM .pbf files was done via filtering per tags (which is similar to an attribute column).

There were two main challenges while filtering: 1) in a .pbf, buildings are not separated from other polygons (e.g., land use) as specific datasets, 2) tag values that can be used for filtering are noisy and incomplete, as OSM mappers are free to use any value of their choice including none. We followed the most common approach, which is to request any non-null value in the building tag/column using a wildcard: building = *. Most values in this column are either *yes* or indicate the type of the building, e.g., house or commercial. A small share of buildings may not have a value in this column and are lost, but adding any other tag, e.g., building: use = * OR amenity = * without requiring a value for the building tag, led to the inclusion of non-building polygons, e.g., district boundary or land use polygons. These would then need to be excluded, which is not trivial. Our approach is conservative in the sense that it prevents true negatives while false positives could only arise from erroneous building tag values, which are expected to be very few.

### Data harmonization

We transformed the retrieved files and harmonized the file formats, the *geometries*, the *attributes*, introduced a common administrative sub-division and a unique identifier (ID) scheme for the whole database. Refer to the lower panel of Fig. 2 for a visualization of the data harmonization steps within the workflow.

#### File formats

We encountered seven different file formats (see input−dataset−metatable−v0.1.xlsx), which we all converted into a single format, see Data Records section. All the file format conversion code and instructions can be found in the repository under /database/preprocessing/1−parsing and in the module preproc.parsing.py^29^.

Parsing.gml and.xml files was made complex by the little support for these formats in high-level Python libraries or desktop GIS tools. In addition, despite the existence of standards for .gml encoding, almost all datasets had specificities in naming relevant elements (the basic building blocks of a XML-type document used as a container to store information), thus requiring a versatile parser. We developed our own parser that retrieves, from any of the .gml and .xml files encountered, building *footprint geometries*, IDs and *attributes* of interest (see code repository^29^, in the module preproc.parsing.py).

Next to.gml and.xml, the parser also handles Shapefiles (.shp) and OSM’s.pbf files by directly reading in *geometries*, IDs and *attributes* using the Python library GeoPandas^36^. For a few files, *attributes* were given as separate tabular files (.csv or .dbf), which we matched to the building *geometries* by ID. Finally, a few files were available as .SQL or.sqlite files or database dumps, which we parsed using PostGIS.

#### Geometries

Harmonizing *geometries* included three main parts: extracting a *footprint* polygon for each building geometry, reprojecting to a single coordinate reference system (CRS) and cleaning the *footprint geometries*.

As the datasets came with *geometries* either in 2D, 2.5D or 3D, we converted all building *geometries* to 2D *footprint* polygons only. We did this for two reasons. The main reason was to simplify the parsing and the structure of the database with a unique building element represented as one row per building. A second reason was to harmonize to a certain extent the representation of buildings in the database. The height dimension was conserved for all 3D buildings via the attribute height, see below. Thus, the eubucco v0.1 adopts a so-called 2.5D representation of buildings, also known as LoD1, as the *footprint* can be extruded using the height value. We acknowledge a voluntary loss of information as the LoD2 datasets contained more details about the roofs that was not conserved in this first version of the database or as certain datasets contained several *height attributes*. Users can find the original data LoD of each input dataset in input−dataset−metatable−v0.1.xlsx. To extract footprint polygons, we used the semantic information when available in the.gml elements, e.g., bldg: GroundSurface or as an attribute in Shapefiles. Otherwise, we dropped the *Z* dimension and projected all building 3D point coordinates onto a 2D (*X, Y*) plane. The datasets also came in 34 different CRS – with some in degrees and some in meters. While a country- or region-specific CRS reduces the bias in the *geometries* due to the projection, a large number of them generates overhead when handling several or all of them together. Therefore, after the initial parsing of the datasets, we reprojected all building *geometries* to a single EU-scale CRS in meters: ETRS89 (EPSG: 3035).

Finally, as the datasets were produced by various different actors and methodologies, sometimes unknown, there were potential differences in building *geometry* definitions, precision and quality. It is out of the scope of this study to comprehensively assess and harmonize these dimensions. We kept the *footprint geometries* mostly identical to the initial datasets. The main alteration of the *geometries* was to harmonize the geometry type to polygon, by converting multipolygons to polygons. We also ran several geometry cleaning steps to detect invalid, empty and null *geometries* and fixed or if not possible filtered them. For specific details, refer to the module preproc.parsing.py^29^.

#### Attributes

**Fig. 3:**
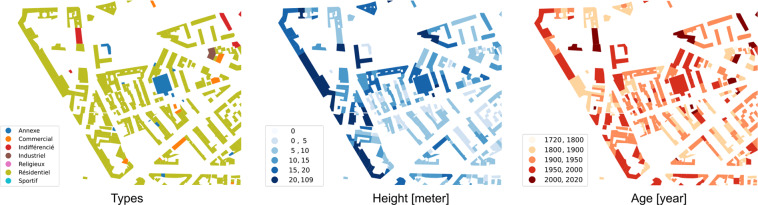
Illustration of the attributes present in eubucco v0.1. The three maps represent buildings *footprints* and the buildings *attributes* present in the database – *type*, *height* and *construction year* – for an example neighborhood in Paris. While the *footprint* shows the urban morphology of the neighborhood, the *attributes* enable to distinguish further contexts.

We harmonized values for the three *attributes* of interest (*height*, *type* and *construction year*) and ensured that a single ID value was available for every building. See Fig. 3 for an illustration of the attributes. We chose *height*, *type* and *construction year* specifically for two main reasons. First, we wanted to limit the number of attributes with low number of values and have the same attributes in all regions for consistency. The selected three attributes are the most populated attributes in existing building datasets in Europe. Additionally, prediction algorithms that can infer missing values for these attributes using machine learning are available in the literature^37–39^ and could be immediately leveraged in a future version of the database. Second, this set of attributes is of high relevance for many use cases, for example energy modeling, see Usage Notes. Future iterations of this database will aim increase the completeness of the initial set of attributes and try to expand to new attributes such as roof type or building materials.

#### Heights

For buildings *heights*, it was not possible to fully harmonize values with one definition, so we used mixed definitions that are transparently documented in input−dataset−metatable−v0.1.xlsx. Indeed, datasets do not all come with the same level of detail and sometimes do not state which height definition is used. The *height* of a building is most commonly defined as the difference between the ground and the highest point of the construction (which can be a chimney), the highest or the lowest point of the roof main structure, e.g., the eaves, or as a percentile of the point cloud generated by the aerial sensing of the building^40^. Those definitions may be similar in case of a flat roof, but possibly lead to large differences in case of steep pitched roofs. Future iterations of the eubucco database would ideally contain a roof type, inclination, orientation, and a estimation of the difference between lowest and highest roof point^41^.

To retrieve building *heights* from the datasets, we either parsed them directly as a single value when available as a .gml element or as a column in a tabular file, computed them as the difference between two values such as ground and maximum building elevation when only such information was provided, or computed them directly using 3D *geometries*. When possible, for example in the case of LoD2 models with semantic information on roof elements, we favored the definition using the lowest point of the roof structure, as it has the most relevant interpretation for use cases interested in estimating living space, e.g., building energy demand models, which we decide to favor in this version. Users interested in other height definitions can modify this aspect in the parser. In cases where only floor information was available, in Spain, Cyprus and for many OSM buildings, we multiplied the number of floors by a floor height of 2.5 m. This fixed value should only be considered as a proxy given that floor height (including the floor-to-ceiling height and additional thickness of the ceiling construction) varies across buildings typically between 2.5 and 4 m^42,43^. It is likely to be an underestimation of the actual height in many cases. The value here also does not include potential roof height and should be interpreted as ground-to-eaves height. Future versions of this database will aim to improve on this aspect.

#### Types

Building *types* also came with different levels of detail across countries. To harmonize *types* while conserving the original level of detail, we used two *type attributes*: type_source (the *type* in the original dataset) and type (harmonized *types*). For the harmonized *types*, we classified buildings into two groups, residential and non-residential, as this was the simplest, yet valuable distinction that can be made.

We manually mapped each type_source value to a type value, creating one matching table per dataset or sometimes one table several datasets when similar codes coming from a cadastral standard were used across datasets, in Germany and Italy. Those tables are available as type_matches−v0.1.zip, see Data Records. When *types* where not available in English, we translated them from their original languages into English using the software deepl.com. We classified mixed-use buildings as residential following the intuition that many mixed use buildings host commercial activities on their ground floors and are used for housing in more than one floor above, making the residential use the predominant use overall, but this may be incorrect in some cases. In cases when the use *type* is ambiguous, e.g., ‘civil building’ we classified the *type* as unknown.

In government datasets, *types* could be retrieved as.gml elements or as a column in tabular files. In some cases, *types* contained strings with semantic information that could be directly used, in others, codes were provided and it was necessary to locate a matching table and replace codes with their semantic counterpart. In OSM, we used the *types* that could be found in the tag building. We removed the ‘yes’ entries from type_source which then corresponded to ‘unknown’ in type. There was an extremely large number of *types*, but we only kept the 53 most common ones, which were used in at least 10,000 buildings and in total account for >99% of all values. We allocated those most common *types* to the residential or non-residential type values, while marking remaining ones as unknown.

#### Construction years

The construction year is assumed to be the year of the end of construction. This information is explicit in a few datasets but often not available. For the sake of conciseness for the variable names, the variable for *construction year* is age in the database, as this is also done in other studies^38^, although there is slight semantic difference. To harmonize building *construction years*, we retrieved only the year from available values. The original value sometimes contained longer formats, e.g., ‘year/month/day’. In government datasets, construction years could be retrieved as.gml elements or as a column in tabular files. In OSM, construction years could be found in the start_date tag. Here, we filtered all values that did not contain relevant numerical information enabling to identify a year.

#### Administrative boundaries

Another major harmonization step was to use a consistent administrative sub-division to enable queries of the database at the country, region and city level. This step was needed as most government datasets and OpenStreetMap do not contain this information for all buildings. We used the Database of Global Administrative Areas (GADM) in its version 3.6, which provides the administrative boundaries of all countries in the world as a single dataset with their names and boundary polygon. We performed a spatial join to match the buildings to their administrative boundaries, as well as region and city name. When a building was located on the boundary between two cities or regions, we allocated it based on the larger area of intersection. The relevant code and details for this step can be found in /database/preprocessing/2−db−set−up/ and in the module preproc.db_set_up.py^29^.

GADM contains several sub-division levels from 0 (country) to 5 (usually district, understood as subdivisions of municipalities). The number of levels varies across countries and the specific meaning of the level also depends on each country’s internal administrative structure. For all countries, we used the level 0 for country and the level 1 for the region-level boundaries. For the city level, we analyzed for each country the boundaries and made a decision on which level to choose. The levels chosen range from level 1 (in the smallest countries like Cyprus where no lower levels were available) to 4 (in larger countries like France). We acknowledge that such grouping may lead to different city definitions across countries, yet we provide a clear overview of which level we chose in gadm−city−levels−v0.1.csv. For example, in rural areas in the Netherlands, the lower level available corresponds to a grouping of several villages, while in Germany those are typically considered as individual cities.

We had to handle city name duplicates for cities with many occurrences, e.g., Neunkirchen, occurring in the German states Baden-Württemberg, Bayern, Nordrhein-Westfalen, Rheinland-Pfalz and Saarland. If these were located in different regions, we renamed the city <city> (<region>). If two cities with the same name were present in the same region, we added indices at the end of the city name to make them uniquely identifiable.

#### Unique identifier

Given the various dataset-specific ID schemes, we had to introduce a harmonized ID scheme, which ensures that each building can be unambiguously identified. We used codes provided in GADM to do so. Our IDs take the form ‘EUBUCCO version identifier – GADM identifier – city-level building identifier’, where the GADM identifier corresponds to the GADM levels existing between country and city level (1, 2, 3 or 4). For example, v0.1−DEU.14.5.4.1_1−1222 is a building from eubucco version 0.1, located in Germany in the fourteenth state, fifth district, fourth local city cooperation group, first city in the GADM code scheme (the ‘_1’ at the end is common to all codes in GADM), and is the 1222^th^ building from this city in our database. Most datasets contained unique building identifiers, which we preserved, however a few did not. In such cases, we created an ID field for the dataset marked by the abbreviation id and ascending number, e.g., id1, id2, etc.

#### Outbound licensing

The final aspect that we harmonized, as much as possible given incompatibilities, was the licensing. Out of 50 datasets, 3 were in the public domain, 41 required only attribution, 2 had an additional share-alike requirement, 2 required a non-commercial use of the data, and 2 did not allow the redistribution of the data. The inbound license of each input dataset used in eubucco v0.1 can be found in input−dataset−metatable−v0.1.xlsx.

One main constraint was the fact that OSM used a share-alike license, ODbL, which requires that any redistribution of the database has be done under the same license. To maximize the number of datasets under the same license, and to facilitate further integration between OSM and government data, we decided to apply ODbL to all possible datasets. By doing this, we could harmonize the license for 46 out of 50 datasets, which represent more than 95% of the buildings in the database. We acknowledge that this choice makes the license more restrictive than some inbound licenses and limits the usage of the relevant data, but it also ensures that any downstream re-use of the data has to remain open access.

For the other dataset whose license had a share-alike requirement but not ODbL (namely Prague, licensed under CC−BY−SA) and for the one dataset that had a non-commercial use requirement (namely Abruzzo, licensed under CC−BY−NC), we redistribute the data under the original license. Finally, for the datasets whose license did not permit redistribution, namely Wallonie and Malta, and for Mecklenburg-Vorpommern where the redistribution conditions were unclear, we did not include them on our repository but provide code to enable users to reproduce the workflow we performed for these datasets, so that they can easily add them to the database for their own usage.

The datasets included on the repository that are not licensed under ODbL are indicated in the repository’s README and their license is also added to their respective file names, i.e., the file name is v0_1−ITA_1−OTHER−LICENSE−CC−BY−NC.zip for Abruzzo and v0_1−CZE_11−OTHER−LICENSE−CC−BY−SA.zip for Prague.

## Data Records

eubucco v0.1^28^ contains 202,191,151 individual buildings each corresponding to an entry/row. The database covers 28 European countries – all the EU countries and Switzerland, see Table 1 for the complete country list –, and contains buildings in 378 regions and 40,829 cities. In addition to the main files containing building-level data, we provide additional tables that either provide information on the database content or enable to match additional attributes to the buildings. The dataset is available as 32 files on eubucco.com and referenced on Zenodo (concept DOI: 10.5281/zenodo.6524780, v0.1-specific DOI 10.5281/zenodo.7225259). There are 28 country .zip files for the data distributed under ODbL, 2 .zip files for the areas distributed under a different license, one .zip for additional files and a license file.

### Main files

The core data are available as 30 × 2 .zip files containing either all columns including the geometry as .gpkg or just the attributes as .csv; for a total of ~123 GB zipped for the former and 2.4 GB for the latter.

#### Structure

The core data are split at the country level, resulting in 28 data chunks (plus two additional files from Italy and Czechia, which follow a different license for distribution) that can be concatenated into one single dataframe for all the EU. Each row of the geometry file contains all variables listed in Table 2, while in the attribute file the geometry column is not included.

**Table 2:** Variables in eubucco v0.1.

Variables	type	Definition
id	string	Unique eubucco building identifier based on the version number of the database, the GADM city identifier and an ascending number for all buildings in the city
id_source	string	Identifier from the original dataset (if no identifier was provided the file name and an ascending number for all buildings in the country was applied).
height	float	Distance in meter between the elevation of the ground floor and of a point representing the top of the building (lowest or highest roof point, highest building point,…); see the relevant *height* definition in input−dataset−metatable−v0.1.xlsx
age	integer	Initial *construction end year* of the building (e.g. not the renovation year, if any)
type	string	Usage *type* of the building, based on our classification ∈ {residential,non−residential,unknown}
type_source	string	Usage *type* of the building, from the original dataset, possibly a human-readable type or a code; see type_matches−v0.1.zip for human-readable matching translated in English if relevant
geometry	string	*Footprint* of the building as a 2D *(X,Y)* polygon object projected in ETRS89 (EPSG: 3035)

Note that that in the attribute-only file the geometry column is ommitted.

The goal of this split is to enable at the same time to: (1) read easily the tabular data in any relevant software (including non-geospatial software such as Excel) without having to load the geometry which represent most of the memory footprint, (2) read easily the geometry and the CRS information through standard functionalities of relevant GIS software and libraries. For the geometry file, we chose GeoPackage (.gpkg) as it is an open, non-proprietary, platform-independent and standards-based format developed by Open Geospatial Consortium. Its memory usage, while not the best across all existing formats, e.g. GeoParquet, is reasonable and it was important to choose a format supported natively by all relevant software, which is currently not the case of the most memory-efficient formats. For the attributes, we chose .csv, a simple, versatile and universally supported tabular file format.

#### Variables

The first and second *attribute* variables are IDs. id is the unique building identifier based on the version number of the database (e.g., v0.1), the identifier of the GADM boundary (e.g., TU.3.2_1) and an ascending number for all buildings in the boundary, connected by a dash. id_source is the ID from the original source file.

The following four attribute variables contain information about building characteristics, including the building *height* (height), *construction year* (age), *type* following a residential/non-residential/unknown classification (type) and the type for the input dataset (type_source). In eubucco v0.1, main attributes have a coverage for height, type and construction year of respectively 74%, 45% and 24% of the buildings. For country-level values, refer to Table 1 and for city-level counts, refer to city−level−overview−tables−v0.1.zip.

The last variable geometry contains the building *footprint*. This is a geospatial vector geometry object, specifically a 2D polygon, represented as a series of point coordinates (*X, Y*) in a referential system defined by a CRS, here ETRS89 (EPSG:3035).

### Additional files

We provide several additional files that may be used together with the main database files, e.g., to match attributes or that can give the user an overview of the content of eubucco v0.1:

1. **Metadata table on input datasets** (input−dataset−metatable−v0.1.xlsx): This table contains 38 dimensions that provide users with the main information about input datasets. Specifically, the file contains the input dataset’s
  name and area information (e.g. country, dataset specific area and dataset name)
  meta info (e.g., access date, data owner, license, link to ressource or download approach)
  structure (e.g., file format, breakdown or additional files matched for *attributes*)
  content relevant to eubucco v0.1 (e.g., availability of given *attributes* or LoDs)
  variable names (e.g., ID, construction year, or building element for.gml files)
  and validation information via the number of buildings at three stages of the workflow (after parsing, cleaning and matching with administrative boundaries) together with the losses that occurred and a short explanation in case of large losses
2. **Table on excluded datasets** (excluded−datasets−v0.1.xlsx): This table provides an overview of available government datasets that were not included in this study with a rational why, most often because they were only available at a high cost. For all these datasets we contacted the data owner to ask whether the data were available for free for research; the status of the dataset reflects their answer and a contact date is also indicated in the file.
3. **Database content metrics at the city-level** (city−level−overview−tables−v0.1.zip): The overview files provide 48 city-level metrics for all 41,456 cities, of which 627, mostly very small cities, do not contain any building. The files enable a detailed overview of the database content in term of *geometries* and *attributes* can be used to study patterns across regions and countries and can also be used to identify bugs or outliers. They are provided as a table for each country with the following naming: <country>_overview−v0.1.csv. Each table contains:
  the city ID, name and region
  building counts and footprint metrics including total number of buildings, total footprint area, footprint area distribution, max footprint area, number of 0-m^2^ *footprint*s, etc.
  *height* distribution metrics in relative and absolute terms, including overall metrics, e.g., median and max value, also outliers outside of a reasonable range, e.g., negative values, and metrics by height bins, e.g., [3, 5 (or [11, 15(
  *type* distribution metrics in relative and absolute terms computed for the variable type and describing the proportion of residential, non-residential and unknown building *types*
  *construction year* distribution metrics in relative and absolute terms grouped by *construction year* bins, e.g., [1801, 1900 (or [1951, 2000 (and also counting additional dimensions such as outliers outside of a reasonable range, e.g., negative values
4. **Type matching tables** (type_matches−v0.1.zip): Multiple tables are provided for each relevant dataset or group of datasets (if cadaster codes apply for several datasets in Germany and Italy) as <dataset>−type_matches−v0.1.csv. These tables enable to map the *type* of the raw data (type_source) to the *type* column (type_source) of the database and provide an English translation of the *type* of the raw data.
5. **Administrative code matching table** (admin−codes−matches−v0.1.csv): this table enables to match the GADM code from building ids with its country, region, city and the input dataset per city. If files were split into parts during the upload process, the table informs which cities are summarized in which region part.
6. **Administrative city levels** (gadm−city−levels−v0.1.csv): this table provides an overview of the GADM level that was chosen to define the city level per country.

## Technical Validation

**Fig. 4:**
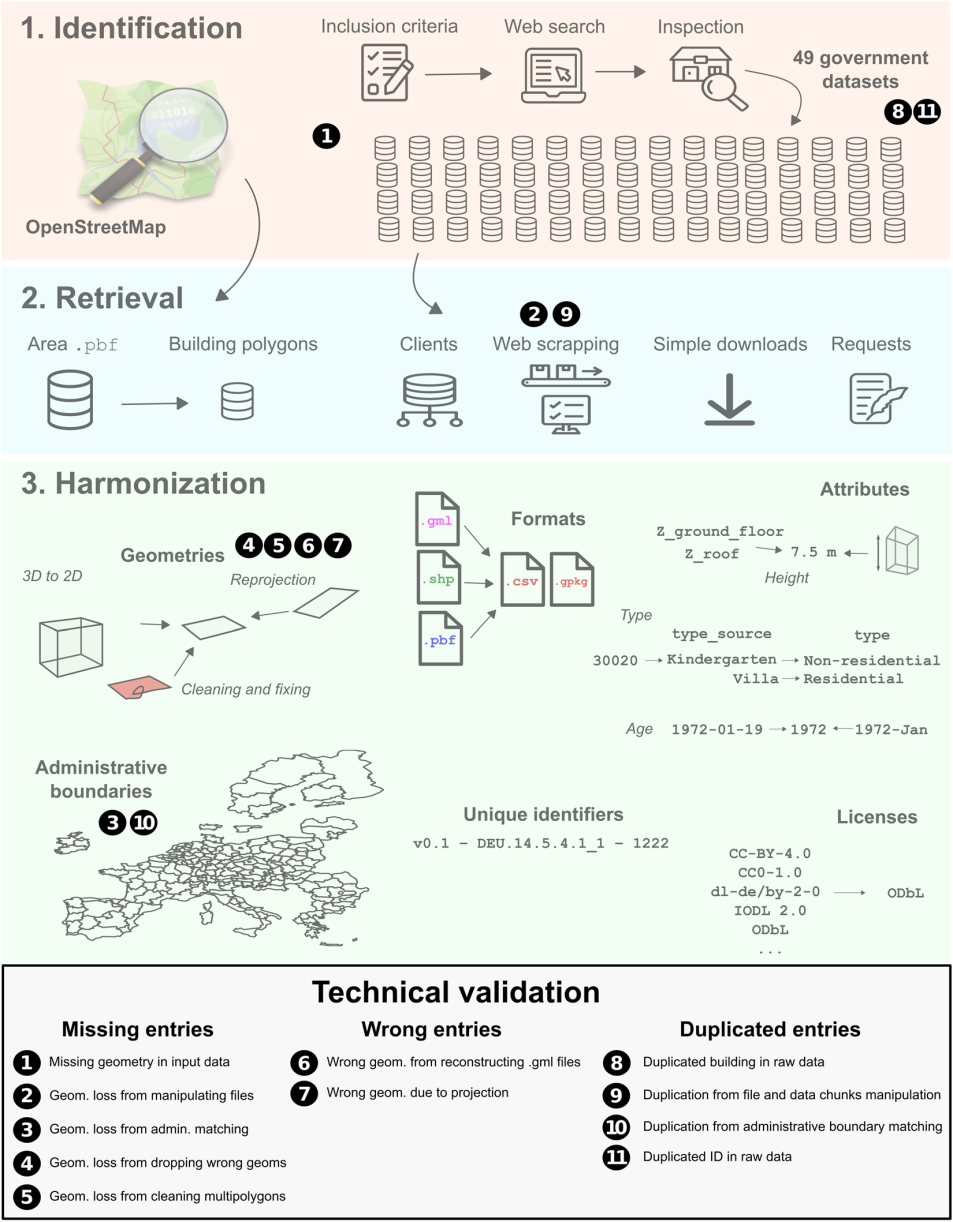
Technical validation workflow.

Our assays focused on guaranteeing the quality of the data in three main areas: 1) ensuring the maximal building stock completeness given the available data; 2) minimizing the number of incorrect data points; 3) ensuring there are no duplicate entries. We performed 11 individual checks, including the analysis of the raw data and consistency checks throughout the workflow to monitor possible data losses from alterations of the data, see overview on Fig. 4. Whenever possible, we implemented automatic tests, such as removing invalid or empty geometries, aiming to guarantee the validity of the data by design.

### Missing entries

We aimed to ensure the maximum building stock completeness given the available data to mitigate biases in overall or local statistics, which also translate into errors in downstream analyses or modeling. Given that the presence of *footprint geometries* is the minimal condition for a building to be included in the eubucco v0.1, their absence equals to missing entries. Missing *geometries* may be due to the fact that buildings are missing in the original datasets or due to manipulations in the pipeline. Data points can be lost due to the introduction of the GADM boundaries or if the content does not meet the requirements of our validation procedure. Whenever we excluded buildings from the original data, we reported losses to provide a transparent assessment of the database coverage, see city−level−overview−tables−v0.1.zip, in the validation procedure columns.

#### Issue 1: Missing geometry in input data

The completeness of OSM depends on the extent of the mapping in each city. In many regions, building *footprints* will be missing in OSM. From our initial analyses, areas with substantially lower coverage are expected in Bulgaria, Croatia, Greece, Hungary, Romania and Portugal. In those countries, our analysis of the overview files shows that for larger cities, the coverage seems to be better than in rural areas. For more details, see city−level−overview−tables−v0.1.zip.

We assumed that the coverage of government data is close to 100%. This is likely to be a few percentage points less for datasets that were produced several years ago. Governments may have omitted a fraction of the buildings for multiple reasons. The provided code for the database allows future data releases to be integrated into an updated version. Future versions of eubucco will aim at more precisely assessing the coverage of input data both for OSM and government datasets.

#### Issue 2: Geometry loss from manipulating numerous files

Some areas were provided as many small tiles, up to 10,000+ for Niedersachsen in Germany or in Poland. We used the overview tables to asses areas with very low coverage or 0 buildings and compared them manually with remote sensing images on google.de/maps, to spot missing tiles. We also inspected manually for all countries that all main cities where present in an order of magnitude that seemed realistic based on their total footprint area. This enabled for example to identify errors due to the webscrapper when many large cities were missing, or also to identify that certain areas had not been included in a dataset. For example, we could identify that in Spain the autonomous communities of the Basque Country and Navarra were missing from the cadaster data and we could use OSM to fill those gaps. This approach, however, does not guarantee that for a city which was cut in several tiles in the input dataset all of them were used, as if some but possibly not all tiles were parsed, the reported metrics may not be identified as suspect.

#### Issue 3: Geometry loss from administrative boundary matching

If the extent of the dataset for an area was larger than the administrative boundary of that area, buildings would fall outside of it. In this case, we decided to drop those buildings to not risk creating duplicates in cases with two adjacent datasets. We monitored the number of buildings before and after this stage to ensure that losses were reasonable. This check also enabled us to ensure that the data was projected correctly and located in the expected region. For more details, see input−dataset−metatable−v0.1.xlsx.

#### Issue 4: Geometry loss from dropping wrong geometries

There are multiple cases in which *footprint* polygons could be considered wrong and that could be assessed automatically at scale. Those included invalid (e.g., self-intersections of lines constituting the polygon), empty (there are no coordinates in the polygon object) and null (the footprint area is 0) *geometries*. For example, we aimed at fixing invalid *geometries*, such as self-touching or self-crossing polygons, by adding a distance of 0 m via the buffer function as proposed in the user manual of the Python package Shapely^44^. After the data cleaning, we dropped all buildings for which the *geometries* could not be fixed and were still invalid, null or empty. We monitored the number of buildings before and after dropping the *geometries* to ensure that the building losses were minimal. For more information on losses after cleaning *geometries*, see input−dataset−metatable−v0.1.xlsx.

#### Issue 5: Geometry loss from cleaning multipolygons

We altered multipolygon buildings to have only polygons in the data, which could lead to losses in some cases. We monitored the number of buildings before and after this stage to ensure that losses were minimal. For more details, see input−dataset−metatable−v0.1.xlsx.

### Wrong entries

Given the size of the dataset, there is a chance that incorrect entries exist in the upstream data or that edge cases caused the workflow to incorrectly parse the data. Here, we analyzed and monitored entries for correctness to avoid generating erroneous statistics or wrong inputs for modeling. We checked that polygons satisfy relevant geometric definitions to be meaningful building *footprints*, and that *attributes* take values in reasonable ranges or string sets (but did not delete unrealistic values).

We checked for invalid, empty and null *geometries* during the initial parsing step and dropped them, see above. Our validation did not include the assessment of the precise positional accuracy of a building (e.g., the building should actually be one meter more to the north), or whether the building may have been demolished since the dataset’s creation.

#### Issue 6: Wrong geometry from reconstructing .gml files

Reconstructing *footprint* polygons from .gml involved to identify and retrieve the correct elements of the xml-tree, in which a *footprint* was often represented as several meshes (triangle polygon) that had to be assembled appropriately. This process was prone to errors as it was different between 2D and 3D *geometries*, between semantically-labelled (e.g., wall, roof and ground surface) and non-semantically-labelled (e.g., *solid* elements), and multiple edge cases had to be accounted for. For example, one dataset (Hamburg) was encoded as individual points wrapped as a mesh instead of having the mesh as the minimal geometry. Therefore, if a wrong reconstruction mode would be falsely selected the footprints may not be reconstructed correctly. To control for that, we checked for the number of invalid geometries after the initial parsing, compared it with the number of individual buildings in the raw file and parsed the dataset again when encountering errors.

#### Issue 7: Wrong geometry due to wrong projection

The projection of the *geometries* could cause two issues in our workflow. First, because of an issue known as *axis order confusion*: in some CRS, coordinates are written in the order (*X, Y*) and in others (*Y, X*), and sometimes it can even be the case across several versions of the same CRS^45^. For datasets provided as Shapefile, this issue was handled directly by GeoPandas. But for the datasets in .gml that we reconstructed manually, we had to check for each dataset whether the reconstructed geometries would lie within the expected bounds. Second, in some rare cases reprojections from one CRS to another CRS in GeoPandas may lead to bugs which transformed coordinates to infinite values. We controlled for these cases by checking for invalid geometries. By matching the parsed data with GADM boundaries, we ensured that we projected correctly as a wrong projection would lead to 0 matches. We controlled for 0 matches using city−level−overview−tables−v0.1.zip.

### Duplicated entries

The last axis of the technical validation was to monitor duplicates. This step is important mainly to ensure that the building stock is not artificially inflated by buildings being present multiple times. It is also important to ensure the building identifiers are unique, in particular to ensure that table matching by ID can be performed safely. There are two main potential sources of duplicated buildings: from upstream errors in the raw data and from manipulations throughout the workflow.

#### Issue 8: Duplicated building in raw data

In rare cases, there could be duplicated entries in the original datasets. We tested for this by checking duplicates on both ID and geometry after the initial parsing, and dropped duplicates when present.

#### Issue 9: Duplication from file and data chunks manipulation

A number of datasets were provided as parts; in such occasions, there were risks that a part is read twice or, when the partitions were created, that some buildings at the end of the part and the beginning of another could be the same. Because some datasets were very large, we also read the data in chunks to keep the memory requirements low throughout the workflow, creating another risk of duplicates. In both cases, we tested for duplicates on both ID and geometry after the initial parsing, and dropped duplicates when present.

#### Issue 10: Duplication from administrative boundary matching

Many duplicates could be created when matching buildings with their administrative boundary by spatial join, based on the intersection between the footprint and boundary polygons. Because administrative boundaries fully fill the area of a country, there are also always adjacent boundaries except for isolated areas like islands. With the join being used, any building that sits on the boundary between two cities would be allocated to both cities. The alternative to keep only buildings within a city was not desirable as all such buildings would be dropped. To ensure that buildings were only present in one city and not duplicated, we calculated the area of intersection for all buildings located on an administrative boundary and allocated them to one city based on the maximum area of intersection.

#### Issue 11: Duplicated ID in raw data

Duplicates in the raw data may come from errors in the data creation, or also from our misinterpretation of an ID field in the data. Indeed, the selected variable may actually correspond to another identification scheme, e.g., at the district level. In some cases, a same ID may be given to several buildings for a given complex or address. To ensure we had only unique IDs, we checked for ID duplicates after the initial parsing.

If ID duplicates were detected, we first tested if the duplicates corresponded to independent buildings. If so, we added an ascending numbering as a suffix. In cases where a duplicated ID marked the same building with identical attributes, provided as several geometries as in Abruzzo (Italy), Piemonte (Italy) and Netherlands, we merged the building parts. In cases where a duplicated ID marked the same building is provided with several geometries and with varying height values per part, as encountered in Flanders, Belgium, we merged the geometries and took the mean of the height values.

## Usage Notes

To assist users in reusing this dataset for their project, we explain how to use the data provided and illustrate this with an example use case. We describe how to filter part of the data, summarize limitations that users should bare in mind and we point to options for users who may want to address some of these limitations by modifying our approach.

### Practical considerations

Using the data likely involves loading buildings with a geospatial software, visualizing them, e.g., as maps, computing metrics describing their characteristics, and conducting downstream analyses or modeling at different scales. We provide a tutorial for several of these aspects, see getting_started.ipynb^29^.

#### Potential scales of use

The availability of location information about city, region and country for each building enables to select data at different scales. The main goal of this work is to enable work at the scale of the European Union. Users aiming to perform any analysis at that scale can build a workflow to exploit all the data provided, likely by loading the data sequentially, given the volume. Users may want to select a single city or region to conduct a local analysis. Finally, users could also choose to select a given subset of areas across different countries to perform comparative analyses.

#### Downloading the data

**Fig. 5:**
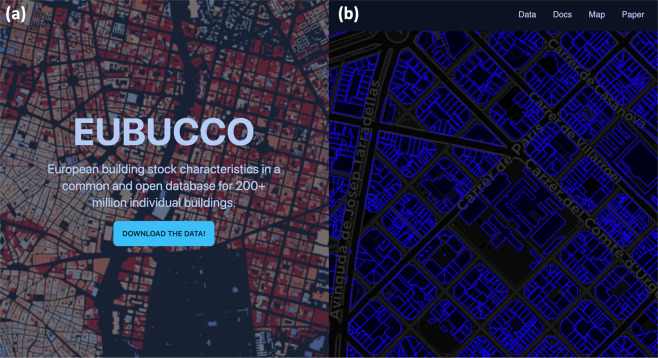
Illustration of the eubucco.com website. (**a**) displays the landing page while (**b**) illustrates the interactive map to explore the building data for the example of Barcelona, Spain.

The data are available on eubucco.com/data, see Fig. 5. Currently, one can download country-level files either manually via the website user interface or via an API api.eubucco.com/v0.1/countries. A few example cities of limited size are available for users who want to explore first a small fraction of the data. In the future, we will provide more refined download options, for example spatial queries.

#### Reading the data

The data can be read by all main GIS software like PostGIS, QGIS or ArcGIS and by the geospatial libraries of the main programming language like Python or R. The full file can be loaded directly as a geospatial layer or a geodataframe. The attribute only-file can be loaded as an attribute table or a dataframe.

#### Visualizing the data

The data can be visualized through maps or by computing and plotting statistics of the *attributes* or of derived metrics. A convenient way to visualize *attributes* is to create choropleth maps, like on Fig. 3, that colors buildings with a color coding corresponding to attribute values. Building *heights* can be extruded to visualize the dataset in 3D,e.g., using the open source framework Kepler^46^.

#### Deriving metrics

An interesting way to enhance the value of the data is to derive metrics that represent particular features of the buildings, individually or aggregated within a given distance or for a city. There are multiple off-the-shelf tools that enable computing various metrics easily. In Python, those include for example the libraries Momepy^47^, GeoPandas^36^, Shapely^44^, PySAL^48^, and OSMnx^49^. One can also find relevant open-source code developed in other studies^37,50^. Refer to the documentation of these libraries for example of metrics and workflows.

#### Integrating EUBUCCO with other datasets

The data can be integrated with various other datasets to conduct richer analyses and modeling. One can match eubucco with geospatial vector datasets, e.g., street networks OpenStreetMap^22^ or land use information from Urban Atlas^51^, to provide a more comprehensive view of urban form. Buildings and streets can for example be analyzed together with tools like Momepy^47^. Geospatial raster data like climate^52^ or population density^53^ can also be relevantly associated, e.g., by aggregating building variables for a grid or using the grid cell value from the raster dataset as a new attribute for the buildings present in the cell. More aggregated statistics, e.g., at the city level, such as greenhouse gas emissions or gross domestic product, can be matched if they can be linked with the boundary polygon of the given area. Then building statistics can, for example, be computed within the same area.

### Example use case: Energy modeling

A concrete example that illustrates how eubucco v0.1 can be used is building energy demand simulations. Energy models require inputs about building characteristics to make predictions about buildings energy demand: statistical models use them as predictive features, engineering models as input parameters. Our data can be used to compute relevant metrics such as the total floor area and the area of each wall for buildings that have a height value. Our data can also be matched with other datasets to derive further metrics, e.g., the solar radiation received by a given wall using gridded climate data or thermal parameters by using the construction year attribute and building typologies datasets. In sum, there is a wide variety of metrics that can be computed from our data for the specific use case of energy modeling, but also for many other such as urban morphology, environmental risk assessment, micro-climate modeling, etc. Building stock data can be used also for material stocks and flow assessments. For instance, Heeren and Hellweg use 2.5D data of Switzerland to determine the amount of construction material in buildings. That allows to determine the current stocks of construction material and anticipate future material flows and opportunities for circular economy^10^.

### Example use case: Climate and disaster risk modeling

Our database can also be used for climate and disaster risk modeling. Such models integrate the natural hazard, the exposure (e.g., buildings), and their respective vulnerability to the modelled hazard^54^. Global climate or disaster risk models often use grid-based exposure data^55^ and large scale (global or continental) vulnerability curves^56^. However, differentiating the exposure and vulnerability on every relevant object has multiple advantages, such as increased spatial accuracy and allowing for differentiated exposure value and vulnerability based on the object characteristics^57^. Our database allows to model buildings as individual objects on a continental scale. Building characteristics such as footprint area, country or number of floors, can be used to differentiate the value and the vulnerability per object, and hence improve current high-resolution risk modeling approaches^58^. Finally, urban overheating negatively impacts health and well-being^59^. Better understanding the relationship between indoor and outdoor temperature on a large scale is crucial to understand indoor-heat risk on a building and floor level and provide decision-makers with quantitative assessments of those risks. Building characteristics such as thermal capacity^60^, the height and orientation are important characteristics to model indoor heat-risk. While certain aspects such as thermal capacity are not directly included in our database, they can be estimated, for instance, based on the building age. Also, variables such as height and orientation can directly be used to model high-resolution outdoor temperature variation of mean radiant temperature^61^ or ambient temperature^62^.

### Filtering options

Users may want to work with a subset of the data based on geography or satisfying certain conditions of homogeneity that are not met across the whole database. Specifically, users may choose to filter for:

1. Only specific countries, regions or cities. This can be done via selecting the relevant country or region file, or by using the admin−codes−matches−v0.1.csv file
2. Only areas with most liberal license, by selecting only the files licensed under ODbL
3. Only areas from government or OSM, by using the admin−codes−matches−v0.1.csv file
4. Only buildings with a given attribute. This can be done by dropping buildings with null values for the attribute.

### Remaining uncertainty

Users should be aware of the limitations of eubucco v0.1 and consider how these may affect the downstream analysis they plan to undertake. As described in several occasions in this document, our attempt was to gather the many fragmented datasets representing the EU building stock and perform minimal harmonization and cleaning to make these data easily usable for local, comparative and EU-level studies. Our attempt was not to perform a detailed assessment of the quality of each dataset nor to ensure its perfect homogeneity. It is complex to map all uncertainties and we leave it to the user to make their own final assessment of the reliability of a given dataset or variable beyond the partial evidence presented in this study. Below, we provide a summary of the main remaining sources of uncertainty that were not fully assessed in this study.

1. The coverage of buildings may be far from 100% in certain areas, in particular in rural areas where OSM was used in countries like Greece or Portugal. For example in Greece, we have ~870 thousands buildings for a population of ~10 million inhabitants in 2019^63^ while we have ~1,9 million buildings in Lithuania for ~2,7 million inhabitant in 2019^63^. This indicates that the coverage in Greece is likely too be overall very low.
2. Attribute values may contain outliers and values outside of realistic ranges, e.g., for *construction years* and *heights* as we did not filter for those in eubucco v0.1.
3. The level of precision of *attributes* (e.g., altimetric precision of the *height*) may differ from a dataset to another. The uncertainty is likely to be higher overall in certain datasets than others, but the uncertainty can also be attribute-specific, e.g., a dataset may have excellent *types* but imprecise *heights*, and it can also be that within a datasets several sources of different qualities were merged.
4. The definition of a building (e.g., grouping or not of several constructions as one building or which types of constructions were included) may differ from one dataset to another.
5. Some buildings may be incorrectly represented, may not exist anymore or may exist but are lacking in eubucco v0.1.
6. Administrative boundaries may not represent the exact same notion of city or region across countries.

### Reproducing the workflow from raw data

Our open-access code repository^29^ and documentation enable users to reproduce our workflow to validate our results or to adapt it to their needs. In various cases, different approaches could be taken and some modeling choices were driven by our specific needs, in particular by the constraints of finding an approach that works across all datasets and that is not prohibitively time-intensive. Users may find other choices more appropriate for their use case, for example if they want to work only with a more homogeneous subset of the data. With the open-access repository, users can obtain source code for handling various steps when working with geospatial data, optimized for large-scale processing, low-memory requirements and fast runtime.

We welcome feedback or suggestions about the datasets present or possibly missing, and about the general approach; in particular, we welcome pull requests on Github. Users wishing to reproduce the study should note that while parsing many of the inputs datasets is possible on a standard laptop, certain steps can be highly memory- or time-intensive. Most of the data processing was undertaken using the Potsdam Institute for Climate Impact Research high-performance computing infrastructure, which provided high memory resources and parallelization over a large number of CPUs.

## Data Availability

All the code used in this study is available on Github as a release: https://github.com/ai4up/eubucco/releases/tag/v0.1^29^. It is free to re-use and modify with attribution under the MIT license.
