# Author Correction: EUBUCCO v0.1: European building stock characteristics in a common and open database for 200+ million individual buildings

**DOI:** 10.1038/s41597-023-02141-y

**Published:** 2023-04-18

**Authors:** Nikola Milojevic-Dupont, Felix Wagner, Florian Nachtigall, Jiawei Hu, Geza Boi Brüser, Marius Zumwald, Filip Biljecki, Niko Heeren, Lynn H. Kaack, Peter-Paul Pichler, Felix Creutzig

**Affiliations:** 1 Mercator Research Institute of Global Commons and Climate Change, Berlin, 10829 Germany; 2 Technical University Berlin, Berlin, 10623 Germany; 3 Independent researcher, Berlin, 12053 Germany; 4 ETH Zürich, Institute for Environmental Decisions, Zürich, 8092 Switzerland; 5 National University of Singapore, Singapore, 119077 Singapore; 6 Norwegian University of Science and Technology (NTNU), Trondheim, 7491 Norway; 7 Hertie School, Data Science Lab, Berlin, 10117 Germany; 8 Potsdam Institute for Climate Impact Research (PIK), Potsdam, 14473 Germany

**Keywords:** Sustainability, Geography, Climate-change mitigation, Climate-change policy

Correction to: *Scientific Data* 10.1038/s41597-023-02040-2, published online 20 March 2023

In this article funding from preproposal phase of the Einstein Center Climate Change and Climate Change Center Berlin Brandenburg for Nikola Milojevic-Dupont was omitted. Also, funding from CircEUlar project of the European Union’s Horizon Europe research and innovation program under grant agreement 101056810 for Florian Nachtigall was omitted. The original article has been corrected.

